# Think Yellow and Keep Green—Role of Sulfanes from Garlic in Agriculture

**DOI:** 10.3390/antiox6010003

**Published:** 2016-12-30

**Authors:** Awais Anwar, Emma Gould, Ryan Tinson, Murree Groom, Chris J. Hamilton

**Affiliations:** 1 Ecospray Limited, Grange Farm Hilborough, Thetford, Norfolk IP26 5BT, UK; murree@ecospray.com; 2 School of Pharmacy, University of East Anglia, Norwich Research Park, Norwich NR4 7TJ, UK; Emma.Gould@uea.ac.uk (E.G.); R.Tinson@uea.ac.uk (R.T.); c.hamilton@uea.ac.uk (C.J.H.)

**Keywords:** garlic, *Allium*, bio pesticides, insecticide, nematicide, potato cyst nematode, diallyl polysulfides, reactive sulfur species

## Abstract

Reactive sulfur species from garlic have long been renowned for their health benefits and antimicrobial properties. In agriculture the subject matter is now gathering momentum in the search for new bio-pesticides to addressing emerging environmental concerns and tighter restrictions on the use of many conventional chemical pesticides. Although the precise modes of action of these garlic-derived bioactives is complex, recent research has provided a number of new insights that deepen our understanding of garlic-derived products, such as garlic extracts and oils. Herein, their activity against various crop-damaging pests is reviewed. In many cases, there seems to be a broad range of activity associated with the sulfur-containing compounds derived from *Allium* species, which manifests itself in diverse insecticidal, antifungal, and nematicidal activities. These activities open a new understanding to develop this natural chemistry as a “green pesticide”.

## 1. Reactive Sulfur Species (RSS) of Garlic and Their Mode of Action

For many centuries garlic and garlic-derived products (e.g., garlic oil) have been widely renowned for their therapeutic and nutraceutical benefits. The antimicrobial effects of garlic are most commonly associated with the broad spectrum of biological activities associated with the sulfur-containing natural products that they produce or that arise during processing [1].

In recent years there has been an increased interest in exploiting the broad spectrum biological activity of garlic-derived natural products to tackle plant pathogens that present threats in food crop settings. This review will focus on the current and potential roles of garlic polysulfide applications in modern agriculture.

**Figure 1:**
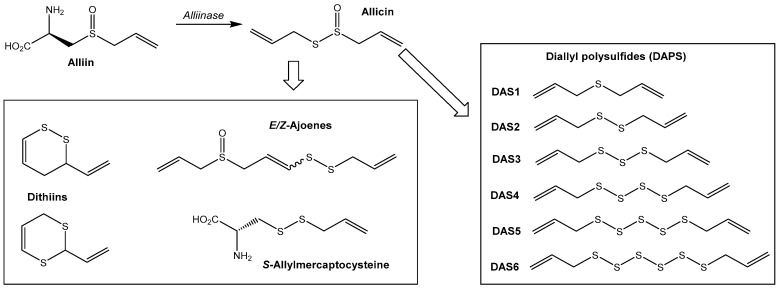
A summary of the key organosulfur compounds derived from garlic. Enzymatic conversion of alliin to allicin occurs immediately upon crushing of garlic. Over time, this degrades to produce an array of organosulfur compound primarily composed of dithiins, ajoenes, *S*-allylmercaptocysteine, and diallylpolysulfides (DAPS). Steam distillation of garlic can be used to produce garlic oils, which are mixtures of DAS1-DAS6 (note: smaller quantities of allyl/methyl mixed polysulfides are also observed in garlic oils/extracts, but their modes of action are analogous to those of DAPS) [2].

The predominant sulfur-containing metabolite produced by garlic is an S-allyl sulfoxide derivative of cysteine called alliin, which accounts for ~1% of the dry weight of garlic. Alliin, which resides in the cytosol of the plant cells, does not itself display any notable biological activity. However, upon crushing, an enzyme called aliinase is released from the cell vacuole and within minutes rapidly catalyzes the conversion of alliin into an unstable thiosulfinate, called allicin. At room temperature allicin slowly decomposes to a stable, but more complex, mixture of other organosulfur molecules such as ajoenes, vinyl dithiins, and diallyl polysulfides (DAPS) (Figure 1). Garlic oils can be produced via the steam distillation of garlic extracts to obtain cleaner mixtures of the DAPS molecules (DAS1–DAS6 plus small quantities of allyl/methl mixed polysulfides). Depending on the source of garlic, the extraction methods, and the age of the extracts, the proportions of these different organosulfur compounds in garlic extracts can vary. It is important to bear this in mind when comparing bioactivity results from different labs. Herein, the primary focus will be on the use of garlic-derived diallyl polysulfides (DAPS).

Garlic oils are made up of a mixture of DAPS molecules whose structures only differ in the number of sulfur atoms that separate the two terminal allyl groups. The number of sulfur atoms ranges from one to six (DAS1–DAS6). The exact DAS1–DAS6 ratios can vary between different garlic oil preparations, but DAS2 and DAS3 are usually the most predominant and account for ~60% of the total DAPS content [3].

When studied as individually isolated DAPS molecules, their biological activity appears to increase with increasing polysulfide chain length (e.g., DAS4 > DAS3 >> DAS2 >> DAS1) [4,5]. However, to date no extensive studies have been performed to see if such trends continue with the longer DAS5 & DAS6 molecules.

**Figure 2:**
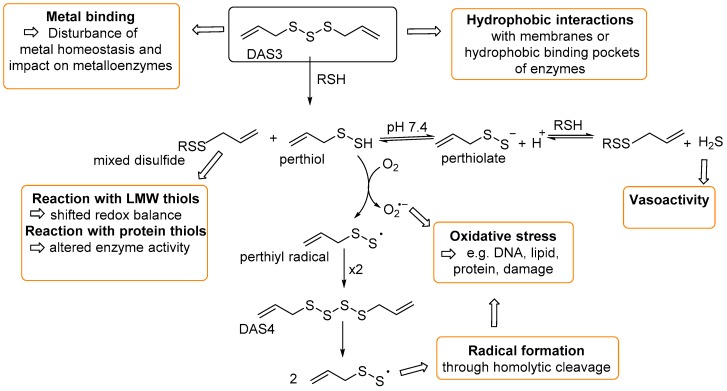
Using DAS3 as an example, possible intracellular reaction pathways of DAPS and their physiological consequences. All reactions described above are possible for DAS3–DAS6, whereas DAS2 is limited to thiol/disulfide reactions with glutathione and protein thiols to form mixed disulfides.

Despite their molecular simplicity, DAPS are able to exert their biological effects via an extensive range of reaction pathways (Figure 2). Once inside cells, DAPS can rapidly react (via thiol polysulfide exchange) with low molecular weight thiols (e.g., glutathione) and protein thiols to disrupt the cellular redox balance and enzyme function, respectively. Such reactions also liberate reactive perthiols, which can subsequently generate superoxide and perthiol radicals [6] that can cause oxidative damage to DNA, lipids, and proteins.

The thiophilic nature of various biologically important metal ions (e.g., Fe, Cu, Zn) means the homeostasis of such metal cations (both free in solution and enzyme-bound) can be perturbed by coordination to DAPS [7]. The lipophilic nature of DAPS means they can also interact with and perturb membrane structures [8].

The multiple modes of DAPS activity severely limit the likelihood of resistance developing in target pathogens. This makes them attractive candidates for pesticide development.

The reader is directed to previous reviews on the likely modes of action of DAPS for a more comprehensive insights into the current state of knowledge in this field of study [9,10].

## 2. Effect of RSS on Different Agricultural Pests

Interest in the development of pesticides based on organosulfur compounds (DAPS) stems from them being food-based bioactives, which should make them more environmentally benign and can obviate the need for pesticide residue limits being implemented during their application.

It is worth noting that the studies presented herein are from different research groups in different parts of the world, using garlic extracts and oils from different sources. The specific activity of these garlic preparations will largely depend on the amount and relative concentration of individual allyl polysulfides that they contain. In most of the reported studies summarized herein, garlic extract was made by ethanolic extraction or garlic was simply crushed in water. Also, no characterization of individual DAPS concentrations in the oils/extracts were provided, which is important as DAPS are the main biologically active molecules within garlic and DAPS of differing polysulfide chain lengths have different efficacies [3]. A recently published article [11] reviewed the effects of pre- and post-harvest conditions on the quality of garlic bioactives produced. A wide spectrum of variables such as cultivar, growth and storage conditions of the garlic, the different garlic extract and garlic oil preparations, and storage methods can all influence the organosulfur composition of the final products.

### 2.1. Insecticidal Effects

The insecticidal market is expected to rise by 4.8% to 3.48 billion USD by 2021 (Market and Market Research data), which makes up roughly 10% of the larger agrochemical sector. This high demand for crop growth and development has therefore applied pressure onto the insecticidal market, which now requires safer and more sustainable products that are biologically benign, cost-effective to produce, and keep the farm operators and consumers safe from harmful side effects.

Garlic preparations have been shown to have insecticidal activity against different life stages of numerous insects that are crop pathogens (Table 1). Garlic extract and garlic oil have been formulated into different pest control products for crop protection for use against various pests. Flint et al., (1995) compared two commercial products, ENVIRepel (Cal Crop USA, Greeley, CO, USA) and Garlic Barrier Ag (Garlic Barrier AG, Glendale, CA, USA), with garlic extract and steam-distilled garlic oil on *Bemisia argentifolii* (whitefly) [11]. It was found that ENVIRepel and Bellows & Perring Garlic oil sprays at a concentration of 2% gave no protection from whitefly; however, a solution of Garlic Barrier Ag of 10% (*v*/*v*) reduced the number of insects, and a 10% garlic extract solution provided the greatest protection [11,12]. Some insects are clearly less susceptible than others, as exemplified by a study of a commercial 26% garlic extract formulation called Alsa (DeruNed B.V., Bleiswijk, The Netherlands), which showed no significant effect in controlling the grapefruit pest *Pezothrips kellyanus* (thrips).

Garlic oil has been shown to possess fumigant activity against the larval sciarid fly *Lycoriella ingénue* with a LC_50_ of 0.87 µL·L^−1^ and various other grain storage insects [13,14]. Comparing the LC_50s_ shown in Table 1, garlic oils have a greater insecticidal effect compared to garlic extracts. This is due to the garlic oils containing greater concentrations of DAPS [15,16].

DAS2 and DAS3 have been shown to have an acute toxicity effect against *C. Chinensis* (bean weevil), where the DAS3 had stronger toxicity than DAS2 and crude garlic oil. It has been demonstrated that DAS3 has strong activity against *S. Oryzae* (rice weevil), *T. Castaneum* (red flour beetle) and *S. zeamais* (maize weevil) [13,14,17]. It has been demonstrated that DAS3 is the strongest fumigant constituent in garlic oil, and is more potent than DAS2 towards *Bursaphelenchus xylophilus* (the pine wood nematode) [14,17,18,19].

In addition to the insecticidal effects, garlic also displays insect-repellent properties. Nowsad et al. (2009) showed a 31% repellency effect of 60% garlic extract against *Dermeste s*sp [21]. Garlic extract has also been shown to have a repellency effect against *T. castaneum* where 95% repellence was achieved [28]. Rahman and Motoyama (2000) used intact garlic cloves, grated garlic, and garlic volatile extracts, in combination with GC-MS analysis, to demonstrate repellent effects against the maize weevil and the red flour beetle were due to allicin and its degradation products [29]. However, Hincapie et al. (2008) [1,3] suggest that methyl 2-propanol disulfide, dimethyl trisulfide, diallyldisulfide, 2-ethylidenedithiane, or *di*-2-propenyl trisulfide and 3-vinyl-1,2-dithiociclohex-5-ene are responsible for the repellent effect after performing gas chromatography to characterize the most effective components against the mite *T. urticae* [24]. This shows that there is uncertainty as to which active components in garlic extract are responsible for its toxicity effects.

Antifeedant activity has also been observed in response to garlic, such as Chaubey (2016) [25] who showed that garlic oil significantly decreased food consumption by *S. oryzae* adults. Garlic has also been shown to have an antifeedant effect against the mulberry mealy bug (*Maconellicoccus hirsutus*) [30].

Another effect of garlic on insects is oviposition inhibition. It has been shown that fumigation of adult *S. oryzae* with a sublethal (40%) concentration of garlic oil causes oviposition reduction [25]. Ho et al. (1996) showed a similar effect where the eggs that were laid from *T. castaneum* and *S. zeamais* in garlic-oil-treated media failed to produce F1 progeny [31]. Oviposition inhibition of *S. cerealella* of 95% (garlic essential oil), 95% (DAS2) and 95% (DAS3) was also achieved at the concentration of 1.5 μL/25 g in a no-choice test [32].

The above findings show the potential for using garlic oil and its major constituents, especially DAS2 and DAS3, as a possible natural insecticide. This is a better option for controlling pests as the currently used insecticides can be highly toxic to humans and non-targeted organisms. Garlic oil and garlic extract have varying effects on different life stages, e.g., *Delium radicum* eggs and adults are more susceptible to toxicity than larvae and different pest species [15], as shown in Table 1. Nonetheless, DAPS chemistry has the potential as a novel insecticide. Further studies for formulation development and application methods are also required to improve the effectiveness and stability.

### 2.2. Nematicidal, Molluscicidal, and Gastropodicidal Effects

Plant parasitic nematodes are causing much more damage annually compared to insect pests. They currently cause annual projected yield losses of 12.3% ($157 billion) worldwide [33]. Most nematode species are free-living and feed upon microorganisms in the water and soil. They inhabit the topsoil at levels in excess of two billion per hectare [1]. The yield losses due to these tiny unseen pests in various countries are enormous. Two prominent plant parasitic nematodes that damage food crops are root-knot nematode (RKN) *Meloidogyne* spp. and the potato cyst nematode (PCN) *Globodera* and *Rostochiensis* spp. *Meloidogyne* spp is known to host 208 genera with 193 countries saturated [34].

Studies concerning the nematicidal effects of garlic (Table 2) often use aqueous or ethanolic garlic extracts, which are toxic to different plant pathogenic nematodes in bioassays [35,36,37]. The effect of garlic extracts on tomato plants showed reductions of root galling, egg masses, and infective juveniles of *M. incognita* and increases in yield upon treatment with aqueous garlic extract. Nematicidal activities of DAS2 and DAS3 are described against the pine wood nematode (*Bursaphelenchus xylophilus*), with DAS3 having a more than 10-fold lower LC_50_ than DAS2 [36]. Distilled garlic oil (GO) was found to significantly reduce root galling after inoculation of tomato roots with RKN (*M. incognita*) [38].

In a recent study [39] the effect of garlic extract was studied on *Meloidogyne incognita*, which significantly affects major food crops all over the world. The experiment showed that garlic extract reduced the galling index, the rate of reproduction of eggs and juveniles J2, and the final population of *M. incognita* by 73%, 80% and 94%, respectively. This is further confirmed by a randomized complete block trial where the effect of different concentrations of garlic oil was observed against *M. incognita*. The experiment concluded that garlic oil (50 µL·L^−1^) was effective in reducing the galling index to 80%, hence improving plant quality.

Similarly, another study using garlic extract on *Globodera pallida* concluded that LC_50_ in J2 nematodes is 983 µL·L^−1^ [38].

It has also been shown that garlic extract has toxic effects against gastropods, including the slug *Deroceras panormitanum* and the snail *Oxyloma pfeifferi* [40,41]. It has been suggested that allicin is responsible for the molluscicidal effects of garlic [41]. This was demonstrated where allicin inhibited the activity of acetylcholinesterase, lactic dehydrogenase, and alkaline phosphatase in vivo and in vitro exposure against *Lymnaea acuminate* [42].

### 2.3. Fungicidal Effects

Estimated food loss based on recent world harvest statistics indicates that persistent disease could lead to losses sufficient to feed 8.5% of the 2011 global human population. The fungicide market is expected to grow by 4.78% to $19.17 billion by 2022 (Market and Market Research). Garlic extracts and oils have shown activity against various crop damaging fungi that cause a huge loss in yield (Table 3). The current challenge posed by fungicide use in agriculture is the rapid onset of fungicide resistance. For example, there are 17 commercial fungicides, which are single-site bioactives that exert their effect by binding to the same ubiquinone binding site of the SDH (succinate dehydrogenase) enzyme. Several of these have been developed in response to the onset of resistance to their SDH-inhibiting predecessors [42]. In contrast, DAPS offer an appealing alternative because of their multiple modes of action.

## 3. The Generation of Green Pesticides

By 2050 the global population is projected to increase by 30% to 9.2 billion, accompanied by an increased demand for food production of 70%, notably due to changes in dietary habits in developing countries towards high-quality food. The reduction of current yield losses caused by pests is a major challenge to improving agricultural production to meet increasing demands.

In the past, synthetic pesticides have played a major role in crop protection programs and have immensely benefited mankind. Nevertheless, their indiscriminate and prophylactic use has resulted in the development of resistance in pests [49], unfavorable environmental side effects, and health concerns.

In the previous decade many synthetic carbamate, organophosphate, and organophthalide pesticides have been banned (Council Directive 91/414/EEC) or are under re-evaluation (Regulation 2009/1107/EC & Directive 2009/128/EC (Information about the Directives can be found on EC website in the area of EUR-Lex (Access to EU Law) [50]. Similarly, in recent years the European Union (EU) has issued a fundamental reform of the Common Agricultural Policy, focusing on respect to the environment, food safety, and animal welfare standards, and demanding that farms implement integrated pest management that should deliver favourable agricultural and environmental outcomes [51,52].

A relevant strategy in the search for new pesticides is the screening of naturally occurring compounds in plants [52]. Plants, as long-lived stationary organisms, must resist attackers over their lifetime, so they produce and exude constituents from plant secondary metabolites that play an important role in their defence mechanisms [53].

Plant secondary metabolites may have applications in weed and pest management if developed for use as pesticides themselves, or they can be used as model compounds for the development of chemically synthesized derivatives. Many of them are environmentally friendly, pose less risk to humans and animals, have a selective mode of action, avoid the emergence of resistant races of pest species, and as a result are more likely to be used safely in integrated pest management [51]. Furthermore, they may be suitable for organic food production.

Within this context, there has been considerable interest in the development of food-based pesticides based on organosulfur compounds (RSS) derived from garlic, onions, and other *Allium* species. As these are food-based natural products, they do not pose problems with residue limits and/or time constraints for the harvest intervals. 

The biological properties against insects, fungi, and nematodes depicted in the previous section are enough to convince us that DAPS chemistry can play a major role in controlling harmful pests without disturbing non-target organisms or having a hazardous effect on the environment [2,49,51,54].

The challenge in developing effective formulations and treatment regimens for DAPS-based pesticides in agricultural settings is understanding the biological constituents and relative concentrations of the bioactive ingredients within the extracts and oils that are to be used. In this regard, one should be cautious when comparing the efficacy of garlic extracts against different pathogens given that the tests have been conducted in different labs (e.g., see Table 1, Table 2 and Table 3), as different garlic extract preparation methods from different garlic sources can significantly influence the composition of the organosulfur bioactives. Many of the previously published studies conducted with crude extract and oils of garlic do not report the composition of biologically active constituents in the test materials.

The development of a pesticide from a natural product requires consideration of important aspects, like a plant extract that shows pesticide activity, the active compound(s) to be identified, and efficacy and dose requirements that need to be tested. The concentration of active(s) in the final product needs to be constant to ensure consistency in product efficacy. Therefore, quality control is essential because the active chemicals in botanicals can vary while processing different batches of plants. Secondly, it needs to be ensured that the formulated product is stable under storage conditions for extended time periods.

One of the driving forces behind the development of new pesticides is the detrimental effects of current chemical pesticides (e.g., neonicotiniods) on important pollinators such as our dwindling bee populations, so it is equally important to test the impact of new pesticides (both chemical and botanical) on bees. A recent study has reported the impact of garlic extracts (Natualho) on honey bees (*Apismellifera*) and shown them to present some toxicity to larvae fed on syrups supplemented with garlic extracts (0.3 mg∙L^−1^) [55]. The same study reports the repellent effects of garlic extracts on adult bees. On the other hand harmful pesticides like neonicotinoid have been found in plants residues. A recent study reported the translocation of these chemicals throughout the plant tissue and found them in pollens, beeswax and the bees themselves. In most cases, the contact acute toxicity (LD_50_-24h) was found to be 4–14 ng/bee [54,56,57]. To date, no studies have been reported that measure the impact on other beneficial insect species.

With this in mind, as with chemical pesticides, the successful employment of garlic oils and extracts in sustainable agriculture requires careful consideration of their formulation and placement (and timing of placement) in order to minimize their risk to such pollinators and ensure that they can be successfully utilized in an environmentally friendly manner.

Various regulatory organizations have now realized the inconsistency of such botanicals and amended their approval criteria. For example, the European Commission has issued new guidance on the proper characterization of botanical extracts before they are issued authorization at the European level [58].

It is worth noting that regulatory approval is quite challenging and expensive. For instance, in Europe the active substance must be registered at the European Commission level before its specific use by national regulatory authorities. For a national approval, the product must be tested for stability, acute toxicity, and aquatic toxicity, and field efficacy data must be generated, which is an expensive task. The regulatory route for approval and use of pesticides in the UK/EU has been reviewed previously [59]. A number of garlic-based products are currently marketed for a range of different agricultural and horticultural applications (Table 4), although many of these have not yet been through the aforementioned regulatory approval process for professional use.

Before any plant protection product can be placed on the market or used, it must be authorized in the member state(s) concerned. Regulation (EC) No. 1107/2009 lays down the rules and procedures for authorization.

The active substance garlic extract is also listed in AnnexI (There are various products on the market claiming the efficacy of garlic for minor uses, e.g. repellent effects. Only one company, Ecospray Limited UK, has so far been successful at registering the garlic extract at the EU level to use as a professional crop protection product) of the EU [60] and is now under consideration for Annex IV inclusion, which sets out the criteria for this material as a low-risk substance having no maximum residue limits (MRL) [50,61].

## 4. Conclusions

Garlic contains a wide range of sulfur agents (RSS) with distinct chemical reactivity, biochemical profiles, and associated biological activities against various crop damaging pests. Based on the published literature, these RSS provide evidence for an “unusual” yet interesting redox chemistry of natural polysulfides in vitro and in vivo. Further investigations will be required, of course, to explore in more detail the various possible chemical and biochemical reaction pathways of DAPS in different organisms and under various circumstances, such as soil types, weather conditions, release into soil, etc.

From a chemist’s perspective, chemically rather simple molecules such as DAS3 and DAS4 seem to be connected with an extensive and quite complicated network of different (bio-)chemical formation and transformation, signalling, and control pathways. Although many of the reactions discussed in this review may ultimately only play a minor role in the biochemistry of DAPS, a combination of several different reactions, rather than just one specific transformation, is likely to be the source of the bioactivity of the DAPS derived from garlic.

Similarly, the in vitro studies reported herein should only be seen as an entry point for wider investigations. Many assays available to date are indirect and often unspecific. Improvements of such assays may hold the key to our future understanding of sulfur redox behaviour in vivo. This applies particularly to assays, which can be conducted in a lab.

Future research may also evaluate their practical use as “green” pesticides and even single components of garlic extract such as DAS3 or DAS4. Either of these pure sulfanes may be ideal candidates for the development of new low risk pesticides.

In summary, considering the chemical and biochemical complexity of DAPS chemistry, it should be no surprise that this area of research provides ample opportunities for future studies at the interface of chemistry, biology, and pest behaviour.

Recent initiatives by the pesticide regulatory departments of European and North American governments have stimulated renewed interest in bio-pesticide technologies to replace toxic synthetic pesticides with more benign natural products. Much progress has been made recently in bringing botanical bio-pesticides to the market and the first well-researched examples of these products are starting to enter significant segments of the EU crop protection market.

However, a concerted effort at formulation development for bio-pesticides by multi-disciplinary teams is still required to optimize yield, efficacy, storage stability, and delivery to enable this technology to evolve further and make a significant contribution to meeting today’s agricultural and societal demands for safe and sustainable food production.

## Figures and Tables

**Table 1:** Efficacy of different garlic compositions on a range of plant pathogenic insects.

Insect	Effected Crops	Garlic Composition ^a^	Life Stage	LC_50_ (mg/L) ^b^	Reference
*Agrotis ipsilon* (Black cutworm)	Strawberries, rice, tobacco, cotton, sugar beet	Garlic oil	Eggs	60	[20]
			Larvae	190	[20]
			Pupae	90	[20]
*Bemisia tabaci* (Silverleaf whitefly)	Tobacco, tomatoes, brassica, cucumber, pumpkin, cotton, melons	Garlic oil	Adults	150	[21]
*Cacopsylla chinensis*	Pears	Garlic oil	Adults	142	[22]
		DAS2	Adults	1104	[22]
		DAS3	Adults	640	[22]
*Callosobruchus maculatus* (Cowpea weevil)	Cowpea beans	Garlic oil	Adults	0.25	[23]
*Delium radicum* (Cabbage root fly)	Brassica	Garlic extract	Eggs	800	[15]
			Larvae	2.64 × 10^5^	[15]
			Adults	1600	[15]
*Heteracris littoralis* (Grasshopper)	Corn, rice, cotton, vegetables	Garlic oil	First instar larvae	670	[24]
*Lycoriella ingénue* (Sciarid fly)	Mushrooms, herbs	DAS1	Larvae	0.25	[19]
		DAS2	Larvae	0.087	[19]
		DAS3	Larvae	0.25	[19]
*Pezothrips kellyanus* (Kelly's citrus thrips)	Citrus fruits	Garlic extract	Larvae	2.6 × 10^5^	[12]
*Sitophilus oryzae* (rice weevil)	Rice	Garlic oil	Adults	0.017	[25]
*Sitophilus zeamais* (Maize weevil)	Maize	DAS3	Adults	5.54	[26]
*Tribolium castaneum* (Red flour beetle)	Flour, cereals	DAS3	Adults	1.02	[26]
*Trichoplusia ni* (Cabbage looper)	Cabbage	Garlic oil	Larvae	3300	[27]

^a^ Production of garlic constitutions varies; refer to corresponding author for details; ^b^ Units changed to mg/L for ease of comparison; refer to corresponding author for the original values.

**Table 2:** Efficacy of different garlic compositions on a range of plant pathogenic nematodes and mollusks.

Effect Type	Crops	Garlic Composition ^a^	LC_50_ (mg/L) ^b^	Reference
Nematicide				
*Melodigyne incognita*	Carrots, parsnips, cotton, tomato	Garlic straw	2000	[39]
*Globodera pallida*	Potato	Garlic extract	983	[38]
*Bursaphelenchus xylophilus*	Pine	DAS3	3	[43]
*Bursaphelenchus xylophilus*	Pine	DAS2	36	[43]
Molluscicide				
*Deroceras panormitanum*(Slug)	All broad leaf crops	Garlic extract	5000	[40,41]
*Oxyloma pfeifferi*(Snail)	All broad leaf crops	Garlic extract	5000 ^c^	[40,41]

^a^ Production of garlic constitutions varies; refer to corresponding author for details; ^b^ Units changed to mg/L for ease of comparison; refer to corresponding author for the original values; ^c^ 30% mortality observed at this concentration of garlic extract.

**Table 3:** Efficacy of different garlic compositions on a range of plant pathogenic fungi.

Fungi	Crop	Garlic Composition ^a^	LC_50_ (mg/L) ^b^	Reference
*Aspergillus flavus*	Cereal grains, legumes, tree nuts	Garlic extract	104	[44]
*Aspergillus niger*	Grapes, apricots, onion, vegetables	Garlic oil	325	[45]
*Botrytis cinerea*	Grapes, strawberry, tomato	Garlic extract	1.3 × 10^5^	[46,47]
*Fusarium oxysporum*	Tomato, legume, cucurbit, banana	Garlic extract	1.6 × 10^3^	[48]
*Penicillium expansum*	Apples	Garlic extract	8 × 10^4^	[47]
*Pythium aphanidermatum*	Soybean, beets, peppers, cucurbits, cotton	Garlic extract	4	[48]
*Rhizoctonia solani*	Soybean, sugar beet, potato, cucumber, rice	Garlic extract	8000	[48]

^a^ Production of garlic constitutions varies; refer to corresponding author for details; ^b^ Units changed to mg/L for ease of comparison; refer to corresponding author for the original values.

**Table 4:** Various garlic-based products on the market.

Product	Use/Crop	Regulatory Status *	Country
Alsa	Repellent	--	Netherlands
Eagle Green Care	Nematicide/Turf, grass	Approved	UK
ENVIRepel	Repellent	--	Italy
Garland	Soil amendment	--	UK
Garlic Barrier	Insect repellent	--	USA
Natualho	Repellent	--	Brazil
NEMguard DE	Nematicide/Carrots, parsnips	Approved	UK, Italy, Cyprus, Greece
NEMguard Liquid	Nematicide/Tomato, peppers, cucurbits Insecticide/Cabbage root fly	Approved	UK, Italy, Ireland
NEMguard PCN	Nematicide, Potato	Approved	UK
Nemater	Soil amendment	--	Netherlands

* Approved at national/EU regulatory level. The “--” sign indicates no approval status found in national pesticide registers.
